# Evaluation and mechanism for outcomes exploration of providing public health care in contract service in Rural China: a multiple-case study with complex adaptive systems design

**DOI:** 10.1186/s12889-015-1540-9

**Published:** 2015-02-27

**Authors:** Huixuan Zhou, Shengfa Zhang, Weijun Zhang, Fugang Wang, You Zhong, Linni Gu, Zhiyong Qu, Donghua Tian

**Affiliations:** China Institute of Health, School of Social Development and Public Policy, Beijing Normal University, 19 Xinjiekou Wai Street, Haidian District Beijing, 100875 China

**Keywords:** Public health, Chronic disease control, Complex adaptive systems, Rural China, Contract service, Family medicine, Multiple-case study

## Abstract

**Background:**

The Chinese government has increased the funding for public health in 2009 and experimentally applied a contract service policy (could be seen as a counterpart to family medicine) in 15 counties to promote public health services in the rural areas in 2013. The contract service aimed to convert village doctors, who had privately practiced for decades, into general practitioners under the government management, and better control the rampant chronic diseases. This study made a rare attempt to assess the effectiveness of public health services delivered under the contract service policy, explore the influencing mechanism and draw the implications for the policy extension in the future.

**Methods:**

Three pilot counties and a non-pilot one with heterogeneity in economic and health development from east to west of China were selected by a purposive sampling method. The case study methods by document collection, non-participant observation and interviews (including key informant interview and focus group interview) with 84 health providers and 20 demanders in multiple level were applied in this study. A thematic approach was used to compare diverse outcomes and analyze mechanism in the complex adaptive systems framework.

**Results:**

Without sufficient incentives, the public health services were not conducted effectively, regardless of the implementation of the contract policy. To appropriately increase the funding for public health by local finance and properly allocate subsidy to village doctors was one of the most effective approaches to stimulate health providers and demanders’ positivity and promote the policy implementation. County health bureaus acted as the most crucial agents among the complex public health systems. Their mental models influenced by the compound and various environments around them led to the diverse outcomes. If they could provide extra incentives and make the contexts of the systems ripe enough for change, the health providers and demanders would be receptive to the transition of the policy.

**Conclusions:**

The innovative fund raising measures could be taken by relatively developed counties of China to conduct public health services. Policymakers could take systems thinking as a useful tool to design plans and predict the unintended outcomes during the process of public health reforms.

## Background

As a result of the post-1979 economic reform, the Cooperative Medical Scheme in rural China began to collapse without the financial support from the collective economy [1]. As a result, more than 95% population lost protection from any form of health insurance by the end of the 1990's [2]. Previous studies showed that lack of health insurance raised the number of rural households living below the poverty line by 44%, since low-income families had to cover high out-of-pocket medical costs by themselves [3,4]. The barefoot doctors, called village doctors after 1985, became unemployed in 1981 and the village clinics were also privatized [5]. Consequently, the village doctors began to charge patients for curative treatment and sell drugs for profit, and shift their focus to lucrative medical services rather than preventative health care [6,7]. However, the outbreak and epidemic of Severe Acute Respiratory Syndrome (SARS) in 2003 exposed the real weakness of China’s public health system [8]. The government realized that the health status of 900 million rural population had considerable impact on Chinese social stability and global health [9], and the fragmentary rural health systems should be re-established to make a quick response to public health emergencies [10]. Therefore, the New Cooperative Medical Scheme (NCMS) was launched in 2003 and covered health insurance for the whole rural population by the year 2010 [11].

However, the health status of China’s population had greatly changed as the economics was dramatically developed over the last decade [12,13], which made the rural health systems in revival too simplistic to deal with [14]. According to the data from the third and fourth China National Health Services Survey, Chinese adults had worse health status in terms of prevalence of chronic diseases in 2008 than that in 2003, particularly hypertension, diabetes, heart disease and stroke. Previous study indicated that chronic diseases rate ratio of rural residents was higher than that of urban residents (the risk-adjusted RR is 1.20 to 1.05), especially for the rural residents, who were over the age of 65 [15]. Rural health systems were required to counter this new challenge in addition to prevention of infectious diseases. To improve the public health care, the Ministry of Health instituted eleven basic items of public health services in 2009, including the establishment of residents’ health assessments, health education, health care of children, maternity patients, elderly and patients suffering from hypertension, type II diabetes and severe psychosis, vaccine inoculation and immunization, response to public health emergencies and assistance in sanitary control [16]. The government also promised to increase the funding for public health annually, from ¥15 (US $2.5) per capita in 2009 to ¥30 (US $4.9) per capita in 2013, and ¥35 (US $5.7) in 2014 [17].

County hospitals, township health centers (THCs) and village clinics constitute the three-tiered health systems in rural China. The basic public health services are undertaken by THCs and village clinics. Specifically, THCs are in charge of the organization work of the public health services, and they can obtain 60% of the funding in accordance with the governmental regulation. Meanwhile, THCs are responsible for the supervision and guidance of the village doctors, and are in charge of the allocation of the remaining 40% of the funding to village doctors, based on the appraisement of their work performance in providing public health services. In other words, the funding distributed to village clinics is the work performance related pay. Unfortunately, the public health related measures have not promoted health providers to supply high-quality services [18], but frustrated the village doctors’ work enthusiasm.

The study conducted in Hubei and Jiangxi provinces showed that the remuneration related to public health services could not offset village doctors’ efforts to the burdensome and time-consuming work, so they still prioritized more profitable medical services [19]. The study in Changzhou County indicated that village doctors felt difficult to provide all the required items of public health services. Most village doctors (92.3%) complained about the “low salary” and “lack of social security” [20]. Our former study in five counties of China also [21] evinced that only 41.3% of the village doctors had pension, and the proportions varied from 2.3% to 83.3% among different counties because the pension were supported by local finance. Moreover, the scarcity and aging of the village doctors was seriously impending and the incentives for the doctor recruitment and retention were in demand [18,22]. Hence, the Ministry of Health launched contract service policy to further improve the rural public health services in 2013 and experimentally implemented it in 15 counties each in one province [23].

The contract service in rural areas can be seen as a counterpart to family medicine which has become a pervasive measure to deliver public health services in countries all over the world [24-28]. After signing the contract with families, the village doctors act as family doctors and need to provide health protection and medical care to the family members individually, although most of them are not qualified general practitioners due to they received their education below college level [21]. In fact, the contract service policy aims to construct a closer relationship between rural residents and village doctors, which can urge village doctors to improve the efficacy of public health services, for example, the early detection of diabetes and hypertension, and other chronic diseases control [23]. On the other hand, village doctors need to sign a contract with THCs and promise to cooperate with them in public health. Village doctors are not members of government officials, and most of them have been practicing privately for decades. The Ministry of Health intends to impel local governments to include village doctors into their administration and provide them with social security and pension by local finance via signing the contract, because the local governments play crucial roles in delivering public health under the decentralization of governmental power [29].

For one thing, decentralization has become a globalized policy in many countries whether rich or poor [30]. The Chinese local governments take charge of health facilities under their jurisdiction and always follow their own ways to pass on policies from central government, thus the amount and quality of public health care vary vastly across the country [29]. For another thing, health care administration is in the charge of at least 16 government agencies. The Ministry of Health is just one of them in low status and mainly responsible for policy making and services providing. Purse strings and the personnel management are in the hand of the Ministry of Finance and the Ministry of Human Resource and Social Security respectively. To resolve the problems related to village doctors’ remuneration and social security would only depend on the finance and administrative capacity of local governments in China [31].

The Ministry of Health has decided to gradually extend the contract service to all rural areas in 2014, on the basis of summarizing the experiences and lessons from the fifteen pilot counties [32]. There are few studies on the evaluation of the diverse outcomes of public health delivery from the contract service pilot sites. Furthermore, the potential factors and mechanism such as how the various contexts and the agents of health systems in different levels, for example the central and local governments, rural health managers, village doctors and patients, influenced the outcomes, need to be explored.

In recent years, health organizations and health reforms have been progressively studied as complex adaptive systems (CASs) [33]. Particularly, a few scholars introduced the concept of CASs into public health evaluation [34,35]. Although there is no real consensus in the definition of CASs [36], it is well-regarded to use a ranging of accepted characteristics of CASs as an analytic framework when an innovative policy is applied in health systems [37].

This study summarized the experiences of public health delivery from four counties, three of which were the contract service pilot site, and the other one was not. The CASs theory was applied as a concept framework to better understand and analyze the outcomes and influencing mechanism of the policy implementation, and the results might offer an approach for the policymakers and executants to perfect their management models and improve the efficiency of public health services in the future.

### Key characteristics of complex adaptive systems

CASs are comprised of numerous agents who may be individuals or corporations. Their interactions and relationships should be seen as the core to understand the systems. Agents act on internal simple rules which may be expressed as instincts, concepts or mental models. They can learn from external environments and adapt to the changes happened in systems. If the contexts around them are ripe enough for changes, in other words, make them feel that the new policy is acceptable, they will feed back actively. Otherwise, they can autonomously oppose to any requirement or just maintain the status quo no matter what happens around them. Moreover, their behavior and passive feedback will influence other agents and the systems. Agents are intelligent and self-organizing. Any control thinking of policy maker is short-sighted, because agents can only be guided or stimulated by incentives rather than be coerced by dictations [38].

CASs is inherently nonlinear, meaning that small changes can have large effects, while seemingly large changes may have little influence on systems [39]. Meanwhile, agents interact in nonlinear fashion and cause events to emerge, and render the whole systems being greater and more complex than the sum of the agents. Therefore, the outcomes derived from interaction and feedback of the agents are unpredictable. Non-reversible processes having similar inception yet lead to different outcomes. For example, the central government implements an innovative policy in several provinces and requires them to reach the same goal. However, the different initial condition [40] and people’s distinctive mental models may guide them to make their own choices along the way, and finally shape different paths and outcomes.

Context dependence is another phenomenon in CASs. CASs are nested systems with fuzzy borders, meaning that each system can be a subset for a bigger one and a supra system for a smaller one, and agents there can exchange information cross-border [41]. Agents’ mental models or other rules in their mind are influenced by their history, culture, values, and norms even illogical or incomprehensible when viewed by others. Likewise, the transition of external environment have impact on agents, and their actions inevitably create ripple effects. As a result, complexity of the systems is generated from the dynamic coevolution.

## Methods

Qualitative case study methods are considered to be suitable for CASs design because they focus on contexts and processes as well as events [42,43]. They are also applicable to answer “how” and “why” questions related to the occurrence of changes. In a multiple-case study, several subjects can simultaneously be dealt with, and the comparison and induction of them can provide more extensive explanations of the issue, and bring people into a more robust conclusion than investigating a single case [44]. Therefore, the multiple-case study approach was used to acquire the circumstantial and complex information about the four study sites, which included three parts: document collection, non-participant observation, and interview with agents in multiple levels, from November 2013 to July 2014.

Four counties in three provinces were purposely sampled depending on their different levels of rural economic and health development. In China, the eastern coastal areas are most developed, the central areas are developed rather more slowly, and the western mountainous areas are the most undeveloped. The origin of NCMS funding is threefold; it comes from the central government, local governments and households. The compensations from central finance are, of course, directed towards the central and western areas [18]. The financing level for NCMS of pilot counties B, C and D, which belongs to three provinces, respectively, locating in east coastline, midland and western mountainous area (Table 1), gradually decreased. We intended to explore the influence of different contexts on the outcomes of public health delivery in the three pilot sites. County A, with the most thriving health and the best economic conditions, is a non-pilot site in the same province as county B. It served as a control site to corroborate whether contract policy had impact on effectiveness of public health services. Based on this, two to four THCs were selected from each county, taking into account the geographical conditions and heterogeneity in health development, by using the purposive sampling. Then, one to three village clinics were selected from each town by a convenience sampling method, and all village doctors in each clinic were interviewed. Concurrently, patients were also interviewed at their convenience when they saw a doctor in the village clinics (Table 2).

**Table 1:** **Background information of the four counties**

**County**	**Geographic location**	**Per capita GDP**	**NCMS per rural capita**	**Rural residents proportion**	**Population served by THCs visited**
County A	Eastern area	¥131169 (US $21420)	¥650 (US $105)	61.67%	479748
County B	Eastern coastal area	¥67933 (US $11094)	¥430 (US $70)	82.76%	161681
County C	Central plain area	¥92725 (US $15142)	¥370 (US $60)	48.99%	47414
County D	Western mountainous area	¥12573 (US $2053)	¥300 (US $48)	80.37%	83600

All the Gross Domestic Product (GDP) data come from the website of the National Bureau of Statistics of China http://www.stats.gov.cn/. Per capita GDP on a country level was ¥41908 (US $6856) in 2013. NCMS per capita of national minimum requirement was ¥350 (US $56) in 2013.

**Table 2:** **Sampling techniques and sizes in different levels**

**Category**	**Sampling technique**	**County A**	**County B**	**County C**	**County D**
Director of county health bureau	\	1	1	1	1
Township health centers	Purposive sampling	3	2	2	4
Village clinics	Convenience sampling	6	6	4	5
Village doctors	\	30	21	6	12
Patients	Convenience sampling	6	6	3	5

Policy documents and reports from local health facilities were collected to extract the information about local management models and some general statistics. The backgrounds of history, culture, population and socio-economics were gathered from the county annals. Non-participant observation in village clinics was used to understand how and when village doctors delivered public health care. The semi-structured key informant interview with 35 people, including the county health bureau directors, the THC managers, and patients in village clinics, was conducted. The total 69 village doctors were interviewed in focus group in the clinic units (Table 2). The interview aimed to explore major agents’ mental models on public health and contract service policy, and their interactions in the public health systems.

Ethical approval was obtained from the Ethical Review Board of Beijing Normal University (BNU) [22]. Verbal consent was taken from all interviewees instead of signing informed consent because of their misgivings about information divulgence. Interviews, lasting for 30 to 60 min, were tape-recorded, transcribed verbatim and typed into Microsoft Word Software without any note of interviewees’ name and accurate address. Likewise, the counties and the provinces they belonging to were anonymous, and quotations in results section just mentioned interviewee’s post.

The thematic analysis approach [45] was used to openly code the data and generate inductive codes [46], which allowed the raw data to be summarized by our categories. Three different sources for data collection helped in triangulating the findings [47]. Information and data from documents could corroborate interviewees’ opinions. Observation and interview could detect information in more aspects to avoid bias of overrated official data. Moreover, interviewing people in different levels could counter the bias derived from one-sided story. We extracted concepts targeting patterns of unexpected events, processes, contexts, relationships and interactions, which were keys to understand the public health systems as CASs [48]. These procedures allowed the authors to identify the relationships among the concepts and ground interpretation on the research questions.

## Results and discussion

### Diverse pathways and outcomes

The three pilot counties had already launched the contract service policy for 8 to 12 months by the time we carried out this study (Table 3). The local governments implemented the national basic public health services in different patterns and led to the divergent outcomes, particularly in terms of the chronic diseases control.

**Table 3:** **Basic situations of village doctors and contract service implementation in the four counties**

**County**	**When contract service implemented**	**Contract signing rate**	**Physician per 1000 capita**	**Pension per month for a village doctor**	**Social security**
County A	Not yet	\	2.99	¥800 (US $131)	Yes
County B	12 months	13.30%	2.20	¥800 (US $131)	Yes
County C	12 months	50.00%	2.72	¥300 (US $49)	Yes
County D	8 months	84.97%	1.60	Not yet	Not yet

Physician per 1000 capita on country level was 2.04 in 2013 according to the data from the website of the National Bureau of Statistics of China http://www.stats.gov.cn/.

In this study, county A did not implement contract service policy. The organization of public health services was rather slack. Little headway was made in health assessments and chronic diseases control. To conduct all public health items would consume gigantic amounts of manpower and material resources of THCs, and the funding for public health could not offset the operating costs. In fact, they skimped the funding deserved by village clinics and overlooked village doctors’ dereliction of duty. A complete health assessment system should contain physical examination and medical record of each villager throughout the year, however, the health assessment files in village clinics were not completed in neither quantity nor quality.

*“Our clinic does not receive any funding for public health. They (THC) asked us to establish health assessments for 3,000 villagers, but we don’t have enough workforces to do that. My two partners and I are over the age of 50. It is laborious for us to input the health data into computer because our computer skills are really poor.” (Village doctor, county A)*

For patients, all of them preferred to go to higher level hospitals for examination and diagnosis of their diseases, and only regarded village clinic as a pharmacy. After all, village clinics had no equipment to measure blood glucose and few doctors here were national licensed. Villagers mistrusted their abilities of medical services. Furthermore, patients were not familiar with the items of public health services they deserved and did not realize the importance of these work, because there was few propaganda conducted by any health organization. The THC managers indicated that only the elderly and the seriously ill patients were willing to participate the free physical examination once a year, health lectures and the quarterly follow-up. As a result, there was not sufficient data about villagers’ health status could be recorded in the health assessments. Few of chronic cases were in the charge of the village clinics.

*“The traffic is convenient now, and almost every family possesses a car. We always go to THC and county hospital to see a doctor. In the village clinic, we only buy some drugs for slight illness such as a cold.” (Villager, county A)*

*“For most villagers, preventive health care is not in demand. They only see a doctor when they have fallen ill. Thus, they are unwilling to cooperate with our work.” (THC manager, county A)*

In addition, the migrant population, who could not participate in the health examination on schedule, was also a factor of the uncompleted health assessments. . The county health bureau director pointed out that the migrant population may become the potential loophole of the diseases prevention and control.

The director attributed the fail to three reasons. First, public health funding and NCMS were managed at the county level and there was not an internet connection among the counties. For this reason, the migrants could not enjoy health service out of their native home. Second, although the ratio of physician per 1000 capita was 2.99 in county A, the highest among the counties visited in this study and exceeding the national average (Table 3), the director thought that the health workforce was in shortage to accomplish all the public health items in high quality. Finally, the funding for public health was insufficient to be shared by THCs and village clinics. Therefore, health providers were lack of work enthusiasm when they were asked to provide public health services.

County health bureau had taken several measures to retain and recruit health workforce. They have insured all village doctors with pension and other social security since 2007, and launched the program about free education for the medical students who signed to become village doctors after their graduation. Two THCs even recruited non- professionals to share village doctors’ workload. However, the effect of these measures could not be seen in short term.

*“The inflexibility of health funding and a large migrant population are national-wide problems……the workforce supplementing measures are slow remedies that cannot meet the urgency nowadays.” (County health bureau director, county A)*

Director also took a gloomy view of the prospect of the contract service policy. The contract, voluntarily signed between village doctors and villagers, did not set any limitation on demanders. Villagers would still follow their inclinations to higher level hospitals, before the medical service ability of village clinics was improved to a more satisfactory level. Even if the THCs discharged their duty on performance appraisal, village doctors could not reach the standard such as a certain contract signing rate or the completeness of health assessment records in such a situation. Ultimately, they would forge the data to obtain the funding.

*“THCs’ assistance and supervision will be operative to enhance village clinics’ service ability in a short while, but now both of them think the potential work is unrewarding. If the contract could not bring village doctors sufficient income and assistance, it is just a dead letter.” (County health bureau director, county A)*

Evidence from the pilot sites county C and D partially verified the negative prediction of the health bureau director in county A, but first to look at the bright side. Following the instruction of superior government, county health bureaus carried out the contract service as an administrative task. The THCs signed contract with village doctors and asked them to persuade villagers to sign contract with village clinics. THCs mainly took charge of health education, propaganda on public health, and management of village clinics, such as to print and disperse leaflets, train village doctors and appraise their work performance. The public health awareness was at least improved among villagers in the process of signing the contract.

However, the contract signing rates in the household units (Table 3) could not represent the effectiveness of public health services. County health bureaus established a supervision system, while the outcomes were counterproductive. They required THCs to reach a certain rate of filling in the health assessments. Thus, the THCs took the rate as one of standards to assess village clinics. If the village doctors could not hit the target rate, their performance-related income will be deducted. Under the circumstances, bogus data of health assessments was spread all over the counties. Several THC managers reflected this problem.

*“Most village doctors are too old to adroitly operate a computer. Even though they collected the data of follow-up, they could not input them into computers by the time of routine inspection. To avoid the income deduction, they have to complete the health assessments in a more prompt way.” (THC Manager, county C)*

*“We need this target rate as our work achievement, so we just open a blind eye on the bogus data.” (THC Manager, county D)*

Meanwhile, the effectiveness of chronic diseases control was not satisfactory neither. Although some village clinics had the authentic information about patients who suffered from hypertension and diabetes, they reported that a large proportion of patients were reluctant to see a doctor in the clinics because they were dissatisfied with the medical conditions there.

*“We are asked to remind the patients of follow-up by telephone, or visit their home to obtain data such as their blood pressure. However, except for the elderly living alone and bed-ridden ones, patients with chronic diseases always go to THC or county hospital to see a doctor.” (Village doctor, county D)*

Directors of the two counties health bureau reported in one voice that most villagers were indifferent to contract service even if they had signed the contract.

*“The public health is financed by the government rather than the residents’ out-of-pocket money. Thus, they don’t care the free services. If they don’t trust the village doctors’ ability, they will not come back to the clinics for follow-up.” (County health bureau director, county C)*

In regard to the low efficiency of public health services, health providers had grievances of their own. Most of THC managers complained about their heavy workload, insufficient remuneration and workforce shortage after the implementation of the contract service policy.

*“We assigned the task of public health to the village clinics and allocated them near 40*% *of the public health funding after signing the contract. However, our staff began to feel dissatisfied because the rest of funding hardly offset the operating costs.” (THC manager,* county *C)*

*“We constantly suffered from workforce shortage. It is getting worse as we settle down to the genuine work of public health services.” (THC manager, county D)*

For village doctors, they were also lack of work incentives because of the limited local financial investment. The issue about their pension and social security were not solved rightly, which was not commensurate with the public health obligation imposed on them. Village doctors in county D had no pension and social security. The pension in county C was ¥300 (US $49) per month, which barely met the minimum standard of living. Social security there was just in initial stage (Table 3). Most village clinics were still operated privately. Village doctors had to commit themselves to providing fee-for-service medical care, whereby they could afford their retirement and indemnity in case of malpractice.

*“Although we have signed a contract with THC, we don’t receive social security as the THC staff. I feel that the government still regards us as peasants rather than doctors in public medical facilities.” (Village doctor, county D)*

The funding for public health brought them the subsidy of ¥5,000 to ¥10,000 annually (varying with the service population in different villages), approximately 13% (county C) to 40% (county D) of their total annual income.

*“If we are devoted to public health services, it will occupy most of our working hours. It means that our income will sharply decline.” (Village doctor, county C)*

The reports submitted by the county health bureaus to the Ministry of Health suggested that more public expenditure should be invested to increase work incentives, recruit workforce and cover social security for the village doctors. Otherwise, the quality of public health care could not be guaranteed.

Contrary to the experiences of the three counties above, health bureau in county B resolved part of the problem by a distinctive funding raising method, and took an important step toward chronic diseases control. The contract service there is a series of pre-paid package services divided into different kinds of chronic diseases including hypertension, diabetes, severe psychosis, chronic obstructive pulmonary disease, malignant tumor and disability. The packages were also divided into high, middle and low classes. Families could choose packages for their family members depending on their types of illness and economic status. The package contains some physical examination items and fee for certain kinds of drugs for one year, extra to the free basic public health services. The contracted villagers buy these packages by approximately two-thirds of the original value, county health bureau will complement the rest of the total expenditure of THCs and village clinics by the NCMS funding.

Take the hypertension package in middle class as an example, the contracted family pay ¥100 (US $16.4) for a hypertensive family member (the original value is ¥150). This patient enjoys test blood pressure once every month (the original interval is once every three months), which is much closer to the international standard of hypertension control [49]; the proportion of the medical charge reimbursement in the village clinics is increased from 50% to 85% which is equal to that of THC; when the village doctors puzzle over any intractable symptom of the patient, they can ask THC cadres to help them solve the problem during the THCs’ biweekly round of visit. If a patient is in a more complex situation and willing to pay an extra ¥30 (¥15 in it is out-of-pocket per visit, the remaining is reimbursed out of NCMS), the village doctors will apply for a long-distance teleconsultation from the specialists in the county hospital.

The upgraded contract service in a preferential price spread rapidly among villagers, and many patients signed the contract and participated in the public health services actively.

*“We don’t need to publicize the public health services and remind patients of follow-up repeatedly. They always clearly remember the date and punctually go to the clinics for follow-up.” (Village doctor, county B)*

For contracted patients, they acknowledged that they could resolve most of their problems in the village clinics.

*“Last month, my blood glucose raised a lot. Village doctors applied for a teleconsultation for me. The specialist adjusted my drugs and solved my problem through the video communication, meanwhile it saved me time and the expense of travelling.” (Villager, county B)*

According to the statistics collected in March 2014, 76.50% of the contracted families bought hypertension and/or diabetes packages charging in middle class. The innovative package services were experimentally implemented in 30% village clinics, so the signing rate in county level was just 13.30%. However, more than 90% patients suffering from hypertension and diabetes in the pilot villages we visited, had been controlled by the village clinics, according to the county health bureau’s report.

THCs took charge of and allocated the package fee as a part of funding for public health. They did not take signing rate as an appraisal standard and never required the village doctors to visit patient home. They only checked the health assessments with the follow-up records through the electronic information system and phoned several patients randomly to authenticate the data. The increase in income related to public health improved THCs’ work incentives and prompted them to help village clinics with their service quality. The village clinics with enhanced ability shared THCs’ duty on public health and reduced their workload. A virtuous cycle was gradually shaping between them.

*“We are willing to assist the village clinics and allocate the funding they deserved, because they can save our manpower and operating costs in public health in the long run.” (THC manager, county B)*

In terms of village clinics, package services increased their funding for public health and the outpatient quantity. The latter one extremely raised their income in registered fee. Therefore, their income did not heavily decrease even if they prioritized the public health services rather than the medical services after the implementation of the contract service. Their total income was, of course, less than that of their previous private practice. However, the medical liability insurance, pension and a full set of social security equal to the treatment of a village official, which were financed by the county government, made them feel that the loss was worthwhile.

*“My previous income was one time more than the income now. However, I had no insurance at that time. Once I was involved into a tangle of medical malpractice, I would dissipate my fortune and go bankrupt overnight. Thus, I consider the status quo better than before.” (Village doctor, county B)*

Nevertheless, the county health bureau director and most of THC managers were still puzzled by the migrant population loophole and health workforce shortage. Some village doctors were also anxious to sign more contracts next year and asked for staffing up.

*“Considering the discount price of the package services, several families have already come to consult us about the contract affairs next year. We have been really busy, and need more workforce to control increasing chronic cases, or we cannot guarantee the service quality in the near future.” (Village doctor, county B)*

### Relationships and interactions between agents

There are a large number of agents with different intentions and functions in the public health systems. The central government makes a series of policies to promote the rural public health services. The provincial and the municipal governments pass on the policies stepwise. These three levels of governments can be regarded as a corporate agent called policy level. The county health bureaus are the authentic executants of public health in the systems. Their decision-making leads to different pathways and outcomes. They can provide financial support, manipulate NCMS and adjust the policies on their own way. THCs and village clinics are health providers who need to maintain their operation and income. If there is no sufficient subsidy for their work, they would rather choose to neglect their duty to avoid economic loss. The rural residents have experienced several rounds of health reforms since 1979. They will not trust any health policy ever, unless they could really benefit from it. They hope to enjoy health service in the neighborhood. However, if the medical conditions in the village clinics is not satisfactory, they would rather go to THCs and hospitals in municipal level to find high-quality service.

**Figure 1:**
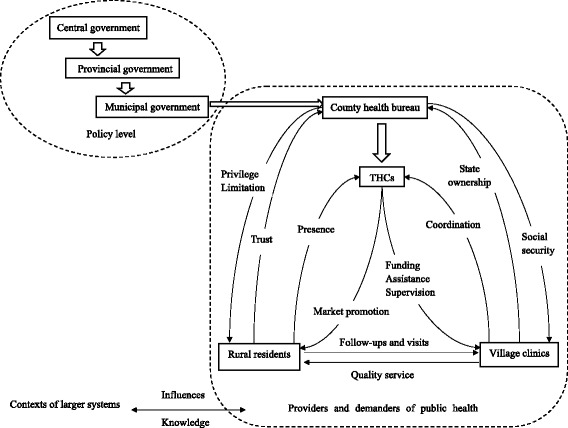
**Major agents and their interactions in the complex adaptive systems framework.** The hollow arrows indicate the official passing on of policies. Linear arrows indicate some consequential interactions or influences. Agents which can be seen as a corporation are rounded by dashed box.

The successfulness of county B mainly stemmed from positive interactions between several agents, which demonstrated the complexity and adaptability of the policy implementation process (Figure 1). First, the patients decided to trust the government again for the sake of the discount they could gain in the contract. Meanwhile, the contract set a limitation on them, that is, if they did not participate in the public health services, the package fee they paid would be in vain. Next, county health bureau provided a complete set of social security to village doctors and delivered a knockout blow to the village doctors’ self-financing practice model. Village doctors genuinely came back to the government management and placed emphasis on providing public health services.

Moreover, cooperative relationship has shaped between the THCs and village clinics. The income from the registered fee in the village clinics and the package fee depended on the demanders’ willingness to pay. Hence, it was crucial for both the THCs and village clinics to provide high-quality services and ensure the contracted patients’ satisfaction. In addition, the package fee did increase their work incentives to some degree. As a result, the THCs stopped skimping on funding for public health, and began to assist and supervise the service delivery of the village clinics.

Feedback loops could emerge between any agents crossing boundaries in a CASs. For instance, the demanders’ positive feedback had impact on THCs and village clinics via the mechanism that demanders altered the environments around the health providers and made the environments ripe for change. Their trust and payment rendered health providers receptive to the contract policy and implemented the public health care cooperatively.

In contrast, the agents in county C and D were lack of incentives to receive any change from the external environments. Moreover, centralized control, such as checking signing rate and imposing a forfeit on village doctors, seriously frustrated health providers and made them resistant to the contract policy. Health providers learned from the innovative policy and behaved adaptively to avoid penalty, for example, some of them used the bogus data to swindle funding. In general, the development of health assessments and chronic diseases control had no essential different with that of county A.

### Agents’ mental models and their contexts

The public health systems were embedded in larger health, economic, historical and political systems of the society. Apparently, there were little links among all these coevolving systems. In fact, they had become a larger context around public health systems which influenced the paths and outcomes of policy implementation through shaping agents’ mental models and providing fresh knowledge for them. That was the reason why agents in different counties conducted various decisions and behaviors. The influences in CASs were, of course, bidirectional between agents and contexts, however this study just focused on the agents (Figure 1).

County A and B are relatively developed eastern regions. Particularly, county B lies in seaboard having several harbors. Both of them are well-known for prosperous business throughout history. Compared to agents in other midland counties, health care executants there had stronger sense of innovation. Furthermore, they preferred to consider cautiously and predict outcomes prior to conducting a policy.

County A foresaw a couple of difficulties in the public health implementation and began to resolve the workforce shortages by several means. County B provided the village doctors with the most comprehensive social security, although its per capita Gross Domestic Product (GDP) is less than county C. It can be seen that the economic status is not the only influencing factor. County B also applied an audacious way to supplement the funding for public health, which became the most crucial incentive mechanism and underpinned the effectiveness in hypertension and diabetes control.

In addition to influenced by the context, agents in county B absorbed knowledge from it. The THC managers introduced market mechanism into public health care. They attracted more population, who were susceptible to chronic diseases, to listen to the health lectures by means of distributing little prizes. They realized that imparting health knowledge to the population between the age of 50 to 65 would have the positive impact on primary prevention and early control of chronic diseases.

*“Our lives are teeming with various marketing tools including doorstep selling, TV advertisement, sending vouchers and so on. Why our health product cannot get a finger in the pie? These little prizes such as washing powder or shampoo can draw more attention of residents to the public health and make our work carried out smoothly.” (THC Manager, county B)*

County C and D are still in the shadow of the threadbare bureaucracy. Their residents take agriculture as their main source of livelihood. Economics and communication in mountainous regions are unprosperous even further. Policy executants and health providers were lack of service awareness. They pursued more short-term profit without long-range planning. The request of signing rate at the beginning of the contract service implementation demonstrated their eagerness to instant success. There was still sense of hierarchy in people’s mind, the village doctors were despised by their superior leaders and received more mandatory orders rather than instructive incentives. Passive promotion methods were applied and issues related to village doctors’ livelihood and rural residents’ right to enjoy public health services had not been solved yet.

*“They (village doctors) are not qualified to be doctors. Public health services are too difficult for them to deliver. In fact, our staff undertakes more workload. We deserve more public health funding than today’s 60*%*.” (THC Manager, county C)*

*“Village doctors are always claiming social security. They are just a group of peasants instead of real doctors, and they have already made much money by privately operating the clinics and worn their barefoot shoes. Whatever else do they want?” (County health bureau director, county D)*

### Lessons learnt

Different from former public health studies emphasizing on a single disease [50,51] or investigating few of provinces, this study unfolded a panorama of public health delivery across three provinces, locating from east to west of China. Although public health care had been funded by the government for five years, the aims to include the village doctors into the government management, effectively control chronic diseases and protect susceptible population, were not realized even under the contract service policy. Distinctively, county B applied an innovative fund raising way and partially solved the problem, also made an initial step toward hypertension and diabetes control. It is indicated that the contract service is just an auxiliary means to prompt public health services. The key element resides in whether the providers and demanders can receive the benefits from the public health care [35].

High-quality health care in a preferential price for the demanders, and work incentives for providers (for example, to increase the income, complete social security, and enhance teamwork between different levels) shaped a virtuous circles and created a more mature context for the changes to happen in county B. In other counties, the public health services were in low efficiency whether the contract program was applied or not. Agents’ resistance and indifference to the public health and contract service arose the unintended outcomes [33], which should be avoid in all countries undergoing health reforms by means of considering the views of all agents [52].

Although there was an argument that the strategies made by the Ministry of Health had decisive impact on the health systems’ ability to adapt [53], we found that the mental models of officials in county health bureaus were consequential to change the systems. They could create different contexts for health providers and demanders under their jurisdictions. Adjustment of the contract policy in local level could accelerate the adaption and diffusion of the public health systems [35]. The mental models of policy executants were not only influenced by the economic status, but also affected by a mixed effect derived from cultural, historical, political and socio-economic environments around them [38]. Therefore, it might be unreasonable to expect every local official can be as audacious, creative and generous as that of county B. However, their successful experiences and advanced measures could be extended to other areas, particular the counties similar to county B in economic status and health development. The potential of NCMS and county finance might be exploited to support rural health systems.

The contract service policy will soon be implemented broadly in rural China. More efforts are also in demand to increase the incentives of both demand and supply sides, resolve the problems related to workforce shortages and the service loophole of the migrant population. The CASs thinking, for the policymakers and executants in all levels, is a useful tool to design plans and predict the unintended outcomes [33,35,54]. Blindly issuing orders and punishing failure will not only waste the health resources, but also frustrate agents to change, and ultimately make public health services into a deadlock. To place emphasis on learning and adjusting policy, introducing incentives and real-time monitoring, creating ripe contexts for agents and making them receptive to changes, will be more effective models in public health reforms, not limited to contract service implementation.

### Limitations

Our study had certain limitations. We used the purposive sampling method to select study sites in provincial, county, township levels rather than the random sampling method. To enhance the sample representativeness, we chose them depending on their heterogeneity in geographical distribution, economic and health development. Moreover, the interviewees were chose from multiple levels ranging from the policy level to the grass-root unit. The sampling quality was sufficient to hit the target in this qualitative study. Second, although the non-participant observation is considered as one of the most objective and impartial study methods, the observers’ existence might influence agents’ behavior and lead to the barriers to the acquisition of profound knowledge. Finally, to comprehensively evaluate the contract service policy and the effectiveness of public health services, quantitative researches are demanded to corroborate the results in the future.

## Conclusions

The effectiveness of the contract service policy to prompt public health depends on whether the providers and demanders can perceive benefits from the public health services. To supplement the funding for public health by a small sum of out-of-pocket money from villagers and the NCMS, provide village doctors with social security by county finance, could be effective approaches to include village doctors into the government management, promote chronic diseases control and enhance demanders’ satisfaction. These measures could serve as a reference for the relatively developed counties of China to increase the incentives of agents in the public health systems. Policymakers and executants could take this CASs thinking as a useful tool to design plans and predict the outcomes in public health reforms. After all, the efficacy and efficiency of the public health services should be enhanced in most rural areas, and several problems such as workforce shortages and the migrant population with the inflexible health funding still need to be properly resolved.

## Abbreviations

NCMS: New Cooperative Medical Scheme
THCs: Township Health Centers
CASs: Complex Adaptive Systems
GDP: Gross Domestic Product
